# The prevalence of diabetes, hypertension and obesity among immigrants from East Africa and the former Soviet Union: a retrospective comparative 30-year cohort study

**DOI:** 10.1186/s12933-016-0392-7

**Published:** 2016-05-05

**Authors:** Yonatan Reuven, Jacob Dreiher, Pesach Shvartzman

**Affiliations:** Division of Community Health, Department of Family Medicine and Siaal Research Center for Family Medicine and Primary Care, Faculty of Health Science, Ben-Gurion University of the Negev, PO Box 653, 84150 Beer-sheva, Israel; Hospital Division, Clalit Health Services, Tel Aviv, Israel; Southern District, Clalit Health Services, Beer-sheva, Israel

**Keywords:** Ethiopian immigrants, East African immigrants, Former Soviet Union immigrants, Ethnicity, Body-mass index, Diabetes, Cardiovascular-risk factors

## Abstract

**Background:**

Previous studies have reported an increasing prevalence of metabolic abnormalities in immigrants who moved from low-cardiovascular-risk regions to Western countries, but little is known about time trends following immigration.

**Methods:**

A retrospective cohort study of immigrants from Ethiopia in east Africa (EAI), the former Soviet Union (FSUI) and native-born Israelis (NBI) over a 35-year period. EAI were divided into three groups by date of immigration. Associations between ethnicity, age, sex and metabolic risk factors were assessed using logistic regression models.

**Results:**

The study included 58,901 individuals (20,768 EAI, 20,507 FSUI, and 17,626 NBI). The multivariate odds ratios (OR) for diabetes were 2.4 (95 % CI 2.1–2.6), 2.1 (95 % CI 1.9–2.2) and 1.5 (95 % CI 1.3–1.7), respectively, for the three waves of EAI immigrations (P < 0.001 for trend) and 1.1 (95 % CI 0.9–1.2) for FSUI. For hypertension, the corresponding ORs were 1.8 (95 % CI 1.6–1.9), 1.4 (95 % CI 1.3–1.5), and 1.1 (95 % CI 0.9–1.2), respectively (P < 0.001) for EAI, and 2.1 (95 % CI 1.9–2.2) for FSUI. For obesity the ORs were −0.5 (95 % CI 0.4–0.6), 0.5 (95 % CI 0.4–0.6), and 0.3 (95 % CI 0.2–0.3), respectively (P < 0.001) for EAI, and 1.2 (95 % CI 1.1–1.3) for FSUI. The prevalence of diabetes in NBI with a BMI of 30 was identical to a BMI of 23.4 for EAI and 28.9 for FSUI.

**Conclusions:**

The prevalence of diabetes and hypertension was higher in EAI and increased over the years, despite a lower prevalence of obesity. It exceeded the prevalence rates in NBI.

## Background

East African immigrants (EAI) from Ethiopia came to Israel in three waves of immigration. The first occurred in 1984–1985 and was known as “Operation Moses”. The second took place in 1990–1991 (“Operation Solomon”) and the third from 2000. In comparison, the vast majority of former Soviet Union immigrants (FSUI) arrived in one wave in 1990–1991, with a smaller number continuing to come through 2001, totalling one million immigrants in all.

Over the first few years following their arrival in Israel a gradually rising prevalence of diabetes was reported in the EAI with relatively low prevalence rates for hypertension and ischemic heart disease. Over the course of 15 years, the prevalence of diabetes and hypertension among EAI increased to about half that of the local population [1–7]. Individuals from the first and second waves of immigration had a low body weight and low blood pressure measurements. [3, 4, 6, 8–14] A steady rise in serum cholesterol levels within 1–3 years after immigration was also reported [4, 5, 9, 12–16]. In contrast, FSUI came from a region with a very high cardiovascular mortality rate. FSU countries have the highest cardiovascular mortality in the world, with rates about three-fold those in Western European countries. Nevertheless, low cardiovascular risk and mortality rates were reported among FSUI who immigrated to Germany and Israel, compared to the general population in those countries [17].

Most previously published data have focused on Black immigrants from the Caribbean or West Africa who immigrated to Western countries over 50 years ago and only some of whom are referred to as EAI. In the present study, we evaluated the pattern of changes over time in the prevalence of cardiovascular risk factors among the three waves of immigration from East Africa to Israel, with up to a 30-year time lag between the first and last wave and compared it with FSUI and native-born Israelis (NBI).

Currently accepted ethnically specific obesity cut-offs have mainly characterized Black populations as a homogeneous group and most studies did not evaluate whether obesity thresholds differ between the various ethnic groups of African origin. Therefore, in this study we compared associations between obesity and the prevalence of diabetes, and determined ethnically specific obesity cut-offs for EAI, which should be tested in other African populations, that equate to those determined in White populations (FSUI and NBI).

## Methods

### Study population

Since the Health Care Reform was passed in Israel (1995), a universal health insurance system provides free access to healthcare services and primary care to all Israeli citizens. This retrospective cohort used a computerized pharmaceutical, medical and administrative database from Clalit Health Services, the largest healthcare provider organization in Israel with about 4.3 million enrollees (about 53 % of the Israeli population and 90 % of EAI in the country).

The original study group included all Ethiopian immigrants over the age of 1 year in order to explore multiple morbidities (cancer, chronic disease). The comparison population was randomly selected from the FSUI and NBI populations, matched by age and sex. Matching was done at the level of the general population. To investigate cardiovascular morbidity, we focused on a sub-group of patients over the age of 35 years. So this sub-group was not completely matched by age. However, we were careful to adjust for age in the data analysis. Thus, the study population included all EAI in three major districts of Clalit Health Services, which were compared with samples of FSUI and NBI among Clalit Health Services enrollees from the same districts, over the years 2002–2012. All participants were enrolled in the Clalit Health Services and had similar access to healthcare services, and a potentially comparable quality of care, although there is some evidence that language and cultural barriers may impede the quality of care provided to new immigrants [18, 19].

### Definitions and exclusion criteria

The country of origin was determined from Clalit Health Services database. EAI were allocated to one of three groups according to the year of immigration from Ethiopia to Israel: during the 1980s, during the 1990s, and those who arrived in the year 2000 or later, similar methodology was used by Kalchiem-Dekel et al. [7]. Since cardiovascular risk factors become prominent starting in the fourth decade of life, a randomized sample of two cohorts (FSUI and NBI) aged 35 years and above, residing in Israel during the years 2002–2012 and matched for age and sex were selected and the NBI were defined as the study reference group. All study groups, including NBI, were of Jewish origin.

Data collected from the computerized database of Clalit Health Services included demographics, anthropometric measures, clinical and laboratory data, hospitalizations, blood pressure, weight, height, BMI, fasting plasma glucose, serum creatinine, total cholesterol, LDL-cholesterol, HDL-cholesterol and triglycerides levels, smoking status, diagnoses (diabetes, hypertension, dyslipidemia, cardiovascular disease) and chronic medications.

BMI was calculated as weight (in kilograms) divided by the square of height (in meters). Elevated total cholesterol, LDL-cholesterol and serum triglyceride levels were defined as higher than 240, 160 and 200 mg/dl, respectively. A low HDL-cholesterol level was defined as lower than 40 mg/dl for males and as lower than 50 mg/dl for females. The LDL:HDL ratio was calculated by dividing HDL-cholesterol level by LDL-cholesterol level. All diagnoses were determined by physicians and recorded in the patient’s electronic medical record. All biochemical parameters were measured in Clalit Health Services laboratories with uniform standards.

A diagnosis of dyslipidemia was based on diagnosis (by the primary care physician, consultants, hospital physicians, etc.) and laboratory values, when total cholesterol, triglycerides, or LDL-cholesterol levels were elevated, or HDL-cholesterol levels were low in accordance with the target values set by the National Cholesterol Education Plan Adult Treatment Plan III (NECP ATP III) guidelines [20]. It should be noted that Clalit physicians document diagnoses from a fixed and uniform list, and when the physician selects a relevant chronic diagnosis, the system attaches a specific code for that diagnosis, which is entered into the database. In addition, the system includes codes for diagnoses from hospitalizations (based on ICD9 codes) and information regarding the purchase of specific drugs, which could be unequivocally linked to specific diagnoses (e.g. insulin).

### Statistical analysis

All statistical analyses were performed using the Statistical Package for the Social Sciences (SPSS, software version 22.0). Baseline data were adjusted for age, and stratified by sex. For continuous variables, mean values, standard deviations, the Student’s t test and the one-way ANOVA test were used. For categorical variables the Chi square test was used and associations between continuous variables were assessed using Pearson’s correlation coefficient. P values for all hypothesis testing were two-sided, and P < 0.05 was interpreted as statistically significant. P values for trend were calculated using year of immigration as a continuous variable in the regression models and were adjusted for age and sex.

Multivariate binary logistic regression models were used to assess the outcomes of diabetes, hypertension, obesity, and dyslipidemia. Separate models were run for each of the outcomes. All subgroups were entered into the same model, which was stratified by subgroup, with NBI as the reference category. An adjusted odds ratio was obtained using a confidence interval (CI) of 95 %. All models were adjusted for age, sex, BMI, smoking, and socioeconomic status.

To compare BMI cut-offs for diabetes, we plotted the age-adjusted prevalence of diabetes against BMI values, stratified by sex and country of origin. Next, we identified the BMI cut-offs in which the prevalence of diabetes in EAI and FSUI, taken separately, was similar to that of NBI.

## Results

The study population included 58,901 individuals above the age of 35 years, of whom 20,768 were EAI, 20,507 were FSUI and 18,787 were NBI. The BMI, glucose level, blood pressure and lipoprotein levels were available for 87, 94, 91 and 92 % of the study population, respectively. The median age of the study population was 51 years (significantly lower in NBI), and 51.8 % were females. The median age at immigration was similar (36 years for EAI and 35 for FSUI), and both EAI and FSUI had a median time of 19 years since immigration (Table 1). The age-adjusted prevalence rates for ischemic heart disease in EAI, FSUI and NBI, respectively, were 1.7, 7.5 and 5.4 % (P < 0.001). The corresponding rates for peripheral vascular disease were 0.5, 2.1 and 1.2 %, respectively (P < 0.001). The corresponding rates for cerebral vascular accident were 4.3, 6.0 and 2.5 %, respectively (P < 0.001).

**Table 1:** Baseline characteristics and cardiovascular risk factors by study groups and sex (age-adjusted rates, individuals >35 years)

	Native-born Israelis (N = 17,626)	Ethiopian African immigrants (N = 20,768)	Former Soviet Union immigrants (N = 20,507)	P value
Age (years, mean ± SD)				
All	50 ± 11	55 ± 16	55 ± 17	<0.001^a^
Males	49 ± 11	55 ± 16	56 ± 16	<0.001^a^
Female	49 ± 11	55 ± 15	55 ± 15	<0.001^a^
Time since immigration (years, mean ± SD)				
All	NA	19 ± 6	19 ± 6	0.414^b^
Males	NA	19 ± 7	19 ± 7	0.054^b^
Female	NA	19 ± 6	19 ± 6	0.433^b^
Age at immigration (years, mean ± SD)				
All	NA	36 ± 17	35 ± 16	0.066^b^
Males	NA	36 ± 18	35 ± 17	0.001^b^
Female	NA	35 ± 16	35 ± 16	0.381^b^
Diabetes (%)				
All	13.4	17.4	14.1	<0.001^a,b^
Males	14.7	16.8	13.4	<0.001^a,b^
Female	11.3	17.9	14.6	<0.001^c^
Hypertension (%)				
All	17.5	18.6	26.8	<0.001^c^
Males	20.1	18.1	26.9	<0.001^c^
Female	15.2	19.1	26.5	<0.001^c^
Obesity (%)				
All	25.6	12.9	28.6	<0.001^c^
Males	24.6	5.1	23.9	<0.001^a,b^
Female	26.6	20.0	32.9	<0.001^c^
Dyslipidemia (%)				
All	55.8	49.9	50.6	<0.001^c^
Males	56.7	48.6	51.2	<0.001^c^
Female	54.9	51.6	50.0	<0.001^c^
Smoking (%)				
All	30.1	7.4	31.8	<0.001^c^
Males	35.8	13.1	44.1	<0.001^c^
Female	24.8	2.1	20.5	<0.001^c^
Total cholesterol (mg/dL, mean ± SD)				
All	190 ± 40	187 ± 45	192 ± 45	<0.001^c^
Males	184 ± 41	188 ± 48	185 ± 46	<0.001^a^
Female	194 ± 39	186 ± 42	197 ± 44	<0.001^c^
LDL cholesterol (mg/dL, mean ± SD)				
All	112 ± 35	111 ± 38	113 ± 40	<0.001^b^
Males	111 ± 36	113 ± 40	111 ± 40	0.163
Female	113 ± 34	110 ± 36	115 ± 39	<0.001^c^
Triglycerides (mg/dL, mean ± SD)				
All	138 ± 80	146 ± 100	139 ± 93	<0.001^a,b^
Males	155 ± 94	160 ± 126	148 ± 109	<0.001^b^
Female	124 ± 63	134 ± 74	131 ± 77	<0.001^a^
HDL cholesterol (mg/dL, mean ± SD)				
All	50 ± 14	48 ± 12	51 ± 14	<0.001^c^
Males	43 ± 10	47 ± 12	45 ± 12	<0.001^c^
Female	55 ± 14	49 ± 12	55 ± 13	<0.001^a,b^
HDL cholesterol/LDL cholesterol ratio				
All	0.43 ± 0.16	0.45 ± 0.18	0.43 ± 0.19	<0.001^a,b^
Males	0.37 ± 0.13	0.44 ± 0.18	0.39 ± 0.72	<0.001^c^
Female	0.48 ± 0.17	0.47 ± 0.17	0.47 ± 0.19	<0.001^a^
BMI (kg/height^2^, mean ± SD)				
All	26.5 ± 4.9	24.6 ± 3.9	27.7 ± 5.4	<0.001^c^
Males	26.9 ± 5.4	23.8 ± 3.1	27.2 ± 4.5	<0.001^c^
Female	26.1 ± 5.4	25.4 ± 4.3	28.2 ± 5.9	<0.001^c^
Glucose lowering medication use(months, median and range)^d^				
All	6 (1–12)	5 (1–12)	6 (1–12)	<0.001^a,b^
Lipoprotein lowering medication use (months, median and range)^d^				
All	5 (1–11)	4 (1–10)	5 (1–11)	<0.001^a,b^

*SD* standard deviation, *NA* non applicable

^a^Ethiopian African immigrants vs native-born Israelis

^b^Ethiopian African immigrants vs Former Soviet Union immigrants

^c^Across all study group

^d^Number months of medication use within a period of 12 months during the year 2012

The age-adjusted prevalence rates for cardiovascular risk factors are listed in Table 1. The prevalence of diabetes in EAI was higher than in FSUI and NBI and highest among EAI females. FSUI females had a higher prevalence of diabetes than NBI, but still much lower than EAI. The prevalence of hypertension in EAI was higher than in NBI, while FSUI had an even higher prevalence of hypertension (Table 1). The prevalence of obesity was significantly lower in the EAI group. However, stratification by sex showed that the prevalence of obesity was significantly higher in EAI females compared to EAI males. Among males EAI had the lowest prevalence of dyslipidemia, while among females FSUI had the lowest prevalence of dyslipidemia. The LDL:HDL ratio was significantly higher in the EAI group. However, stratification by sex showed that the LDL:HDL ratio was significantly higher in EAI males compared to FSUI and NBI males, while EAI females had similar LDL:HDL ratio compared to FSUI and NBI females. Smoking rates were significantly lower in EAI in both sexes (Table 1).

**Fig. 1:**
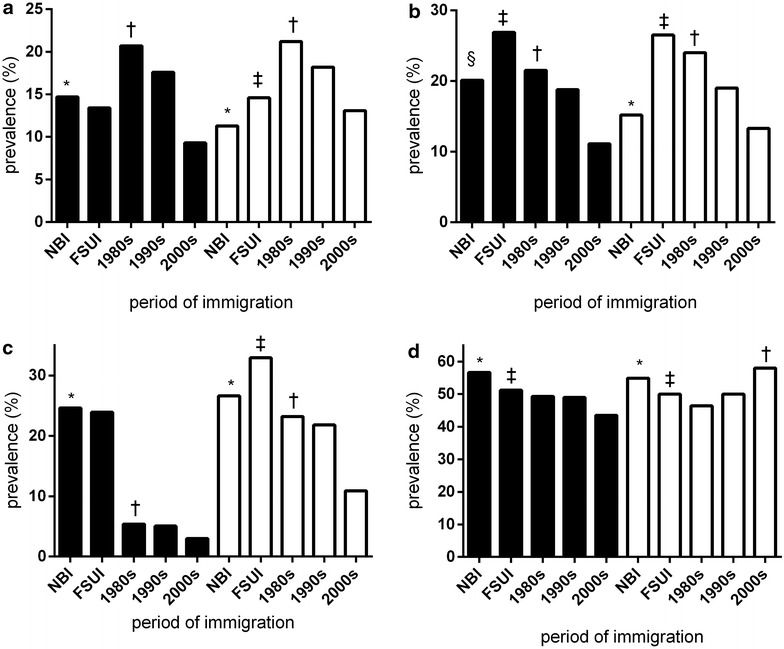
Prevalence of cardiovascular risk factors by sex and period of immigration by country of origin (age-adjusted rates, individuals >35 years). *P < 0.001, NBI vs across period of immigration, within sex ^†^P for trend <0.001, across period of immigration, within sex. ^‡^P < 0.001, FSUI vs NBI, within sex. ^§^P < 0.05, across period of immigration, within sex. **a** Diabetes, **b** hypertension, **c** obesity, **d** dyslipidemia. *Black bars* male; *White bars* female; *NBI* native born Israelis (reference group), *FSUI* Former Soviet Union immigrants

The age-adjusted prevalence rates for cardiovascular risk factors in EAI, stratified by period of immigration and sex, and compared to FSUI and NBI, are depicted in Fig. 1. In EAI the prevalence of diabetes (Fig. 1a), hypertension (Fig. 1b), and obesity (Fig. 1c) in both sexes increased gradually with the time from immigration. Thus, it was highest among those who immigrated during the 1980s, eventually surpassing the prevalence in NBI 30 years after immigration (P for trend <0.001).

**Fig. 2:**
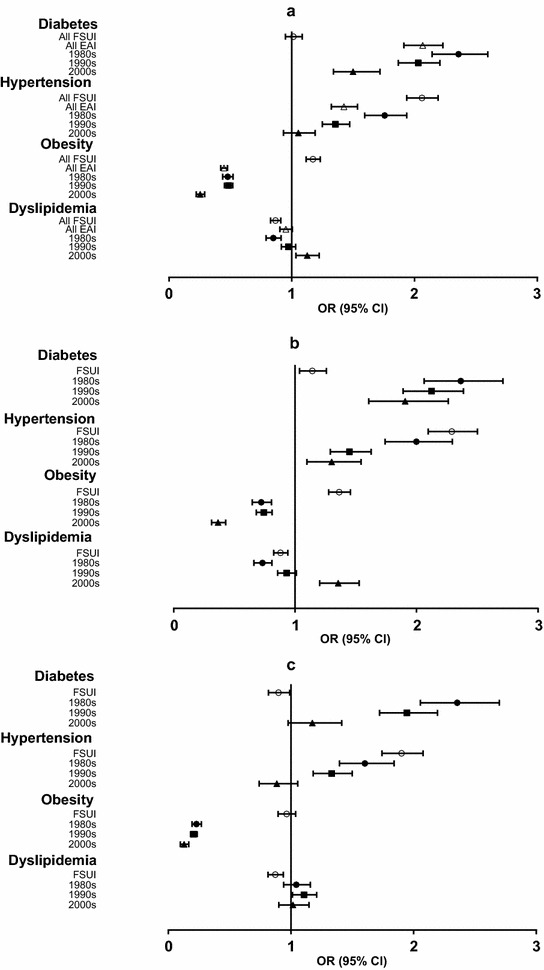
Cardiovascular risk factors by country of origin and period of immigration by country of origin (multivariate logistic regression model). Odds ratios from multivariate logistic regression models of cardiovascular risk factors by period of immigration vs native born Israelis controlled for confounders. **a** All study group, **b** female, **c** male. *White triangle* East African immigrants (all); *White circle* Former Soviet Union immigrants; *Black circle* 1980s immigrants; *Black squares* 1990s immigrants; *Black triangle* 2000s immigrants. All P for trend <0.001 except diabetes in males p for trend =0.003

Age, sex, BMI, smoking, and socioeconomic status were added as confounders to the multivariate analysis models for diabetes, hypertension, obesity, and dyslipidemia (Fig. 2). Multivariate odds ratios (ORs) for diabetes were higher in EAI in both sexes (Fig. 2). However, the strength of the association decreased for recently arrived EAI. Immigrants who came to Israel during the 1980s were 2.36 times more likely than NBI to have diabetes. The corresponding ORs for immigrants who came to Israel during the 1990s and the 2000s were 2.04 and 1.50, respectively. No statistically significant association was found between FSUI and diabetes.

A similar pattern was noted for hypertension (Fig. 2), with significant ORs for female EAI (Fig. 2b) and male EAI who immigrated before 2000 (Fig. 2c). As for FSUI, ORs for hypertension were 2.05 for the entire FSUI sample, 1.9 for males and 2.3 for females. ORs for obesity in EAI were significantly lower for both sexes, regardless of the time of immigration. The ORs for obesity in females who immigrated during the 1980s and 1990s were higher than in those who immigrated after 2000, although still lower than in NBI. In contrast, ORs for obesity in FSUI females were relatively high.

A reverse trend was noted for dyslipidemia, with lower OR in EAI who immigrated during the 1980s (especially females) and higher ORs (in comparison to NBI) in recent immigrants (Fig. 2a, b, respectively).

**Fig. 3:**
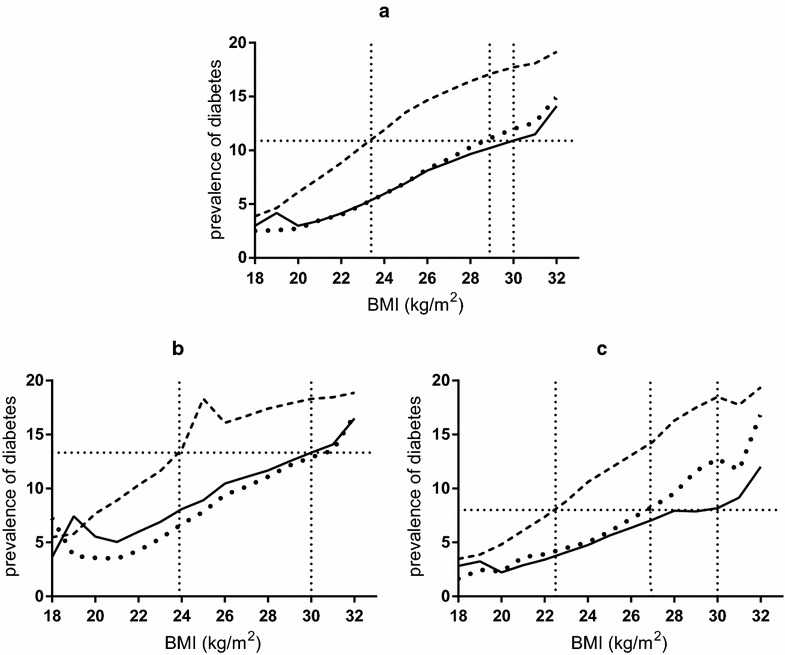
Age-adjusted prevalence of diabetes by BMI, by country of origin. **a** All study group, **b** male, **c** female. *Solid line* diabetes prevalence in native born Israelis (reference group), *dashed line* diabetes prevalence in East African immigrants, *spotted line* diabetes prevalence in Former Soviet Union immigrants

When plotting the association between age-adjusted prevalence of diabetes and BMI (at any given BMI), the prevalence of diabetes in EAI was similar to FSUI and NBI at higher BMI levels (Fig. 3). The prevalence of diabetes in NBI with a BMI of 30 was identical to that of EAI with a BMI of 23.4 and of FSUI with a BMI of 28.9. When stratified by sex, the prevalence of diabetes in NBI females with a BMI of 30 was equivalent to that of EAI with a BMI of 22.5 and FSUI with a BMI of 26.9. The prevalence of diabetes in NBI males with a BMI of 30 was equivalent to EAI males with a BMI of 24.0 and to FSUI with a BMI of 30.9 (Fig. 3).

## Discussion

The results of our study demonstrate ethnic differences in both the prevalence of cardiovascular risk factors and the association between obesity and diabetes in relation to time since immigration of EAI to Israel, a Western environment. The prevalence of diabetes and hypertension in EAI gradually increased over 30 years of residence in Israel, to the point where it exceeded the prevalence in NBI. FSUI had a higher prevalence of obesity and hypertension compared to NBI. Female FSUI also had a higher prevalence of diabetes. These findings were still significant after controlling for confounders, allowing us to focus on the differences by country of origin.

Consistent with previous studies, Black males and females had a higher prevalence of diabetes and hypertension than Whites [21–29]. However, these abnormalities occur at BMI cut-off points that are usually considered normal in Western countries. In the Black population the prevalence of diabetes in subjects with a BMI of 22.5–24 was equivalent to that in obese White populations (BMI >30). This strengthens conclusions done by Kalchiem-Dekel et al. [7]. for the adaptation of different BMI cut-off points for the assessment of cardiovascular risk among Ethiopian immigrant populations. Additionally, this is consistent with previous studies by Stommel et al. and Ntuk et al. suggesting a range of BMI cut-offs from 23–26. These studies did not include a sex-stratified analysis and did not take into account the different origins of the Black population [30–32]. The findings are consistent with studies suggesting that the cut-offs currently recommended by the WHO should be reduced when applied to non-White populations, as reflected in the Harmonized Joint Interim Statement on metabolic syndrome, which also includes recommended waist circumference values for sub-Saharan countries [33, 34].

It is of note that the prevalence of ischemic heart disease, cerebrovascular disease and peripheral vascular disease in EAI was lower than in the other two groups despite the higher prevalence of diabetes. A possible explanation suggested by Valenzuela-Garcia et al. [35] is that even the degree of glycaemic control in diabetes is not always correlated with coronary flow. Similar findings were reported for microvascular complications [36]. Therefore, other unknown factors might be involved.

The results of our study suggest that different obesity thresholds might be adopted for Black Africans as indicated in this present study. This should be tested further in other African populations, as suggested by Barrett Sewali et al. [37] who compared cardiovascular risk factors across six African immigrants groups. It should be noted that a marked degree of genetic heterogeneity exists in Africa, beyond genetic differences between Blacks and Whites, which might bring about a different genetic make-up in Ethiopians compared to African–Americans [38].

Several hypotheses have been proposed to explain why Black populations have an equivalent risk of diabetes at lower levels of BMI. Some researchers attribute this to higher insulin resistance among Black populations due to the deposition of body fat in the abdomen at lower BMI levels. They also contend that the “thrifty gene” inherited from African ancestors enables them to maximize metabolic activity and store calories more efficiently during periods of famine, but predisposes them to metabolic morbidity in times of abundance. Additionally the thrifty phenotype hypothesis should be considered, as it suggests that poor nutrition in early life influence changes in glucose and insulin metabolism and can eventually lead to the development of type 2 diabetes [39, 40]. The “thrifty gene” hypothesis may also apply to the increase in prevalence of cardiovascular risk factors in Black immigrants, together with other factors such as unhealthy nutrition, lifestyle and immigration-related stress [38, 41, 42].

Gillum [42] described six stages of transition from rural tribal society to modernized urban communities that can affect the prevalence of hypertension. Nutritional factors could contribute to the discrepancy in the prevalence of dyslipidemia, since poorer EAI are more likely to consume cheaper food products, which have a higher content of sugar, salt and saturated fats, and a lower content of micronutrients and antioxidants [43]. Differences in physical activity levels (especially among women), hormonal factors, and other environmental factors, might play a role as well. The results of our study are consistent with previous reports on the high prevalence of hypertension in FSUI [44]. We also found a higher prevalence of cardiovascular morbidity, in contrast with several studies that found lower cardiovascular risk and mortality among FSUI compared to the general population. Our study showed that the age-adjusted prevalence rates of ischemic heart disease and peripheral vascular disease in FSUI were four times higher than EAI and about 1.5 times higher than NBI. This finding can be attributed to the fact that FSU countries have the highest cardiovascular mortality in the world with mortality rates about three times that of Western European countries [17].

Our study population consisted of 20,768 Blacks, which is about three times larger than any previous study on BMI cut-offs in a Black population. This allowed us to determine precise ORs and prevalence rates, and to provide a more detailed picture of trends in this population. We also had a unique opportunity to conduct an “experiment of nature,” documenting the effect of immigration, beginning with the first years of immigration with a follow-up that reached as many as 30-years in many cases. Due to these findings we also believe that the “healthy immigrate effect” may no longer be valid [45].

Thus, a major strength of this study was our ability to compare two major immigration groups from very different parts of the world that live in the same country, have similar immigration characteristics, and are treated in the same health care system. While most of the previous studies were cross-sectional in design, the present study was on a retrospective cohort.

Our study has some inevitable limitations. We were unable to differentiate between type 1 and type 2 diabetes. However, the vast majority of diabetes cases is type 2 (90–95 %) [46, 47] and in our study population aged 35 years or above, the proportion of type 2 diabetes is probably even higher. In addition, it should be noted that the waist circumference, which is not routinely measured by Israeli primary care physicians is more indicative of abdominal fat distribution and therefore more relevant for diabetes development. This might explain the occurrence of diabetes at lower BMI levels in Ethiopian immigrants. Therefore, measuring waist circumference, in addition to weight and height, by physicians taking care of Ethiopian immigrants should be encouraged.

Our study adds to the growing body of evidence that Blacks face a greater burden of diabetes and other cardiovascular risk factors at lower levels of BMI, supporting the position that lower obesity thresholds should be applied to non-White populations. Doing this might help improve data collection for studies of migrant health as suggested by Modesti et al. [44], and ensure an equitable approach based on equivalent risk when assessing cardiovascular risk among African immigrant populations [48]. We also believe that BMI levels may have less significance in Black EAI when weighing the importance of nutrition in the treatment of diabetes. Another aspect that should be considered is the therapeutic significance of setting a lower threshold for obesity.

## Conclusion

FSUI had a high prevalence of cardiovascular risk factors including hypertension and obesity in both sexes, and diabetes in females compared to NBI, and their BMI cut-off for diabetes was similar to NBI. In contrast, the prevalence of diabetes and hypertension was higher in EAI compared with NBI, while the prevalence of obesity and dyslipidemia was lower. The prevalence of diabetes and hypertension increased directly with the time since immigration, exceeding the prevalence in NBI. The prevalence of diabetes at any given BMI value FSUI and NBI was similar to that of EAI with a BMI lower by 7 kg/m^2^.

## Abbreviations

EAI: East African immigrants
FSUI: Former Soviet Union immigrants
NBI: native-born Israelis
